# Naloxone’s dose-dependent displacement of [^11^C]carfentanil and duration of receptor occupancy in the rat brain

**DOI:** 10.1038/s41598-022-09601-2

**Published:** 2022-04-19

**Authors:** Yeona Kang, Kelly A. O’Conor, Andrew C. Kelleher, Joseph Ramsey, Abolghasem Bakhoda, Seth M. Eisenberg, Wenjing Zhao, Tyler Stodden, Torben D. Pearson, Min Guo, Nina Brown, Jeih-San Liow, Joanna S. Fowler, Sung Won Kim, Nora D. Volkow

**Affiliations:** 1 Laboratory of Neuroimaging, National Institute on Alcohol Abuse and Alcoholism, National Institutes of Health, Bethesda, MD 20892-1013 USA; 2 Department of Mathematics, Howard University, Washington, DC 20059 USA; 3 Molecular Imaging Branch, National Institute of Mental Health, National Institutes of Health, Bethesda, MD 20892 USA; 4 National Institute on Drug Abuse, National Institutes of Health, Bethesda, MD 20892-1013 USA

**Keywords:** Medicinal chemistry, Pharmacology, Drug discovery, Neuroscience

## Abstract

The continuous rise in opioid overdoses in the United States is predominantly driven by very potent synthetic opioids, mostly fentanyl and its derivatives (fentanyls). Although naloxone (NLX) has been shown to effectively reverse overdoses by conventional opioids, there may be a need for higher or repeated doses of NLX to revert overdoses from highly potent fentanyls. Here, we used positron emission tomography (PET) to assess NLX’s dose-dependence on both its rate of displacement of [^11^C]carfentanil ([^11^C]CFN) binding and its duration of mu opioid receptor (MOR) occupancy in the male rat brain. We showed that clinically relevant doses of intravenously (IV) administered NLX (0.035 mg/kg, Human Equivalent Dose (HED) 0.4 mg; 0.17 mg/kg, HED 2 mg) rapidly displaced the specific binding of [^11^C]CFN in the thalamus in a dose-dependent manner. Brain MOR occupancy by IV NLX was greater than 90% at 5 min after NLX administration for both doses, but at 27.3 min after 0.035 mg/kg dose and at 85 min after 0.17 mg/kg NLX, only 50% occupancy remained. This indicates that the duration of NLX occupancy at MORs is short-lived. Overall, these results show that clinically relevant doses of IV NLX can promptly displace fentanyls at brain MORs, but repeated or higher NLX doses may be required to prevent re-narcotization following overdoses with long-acting fentanyls.

## Introduction

**Figure 1:**
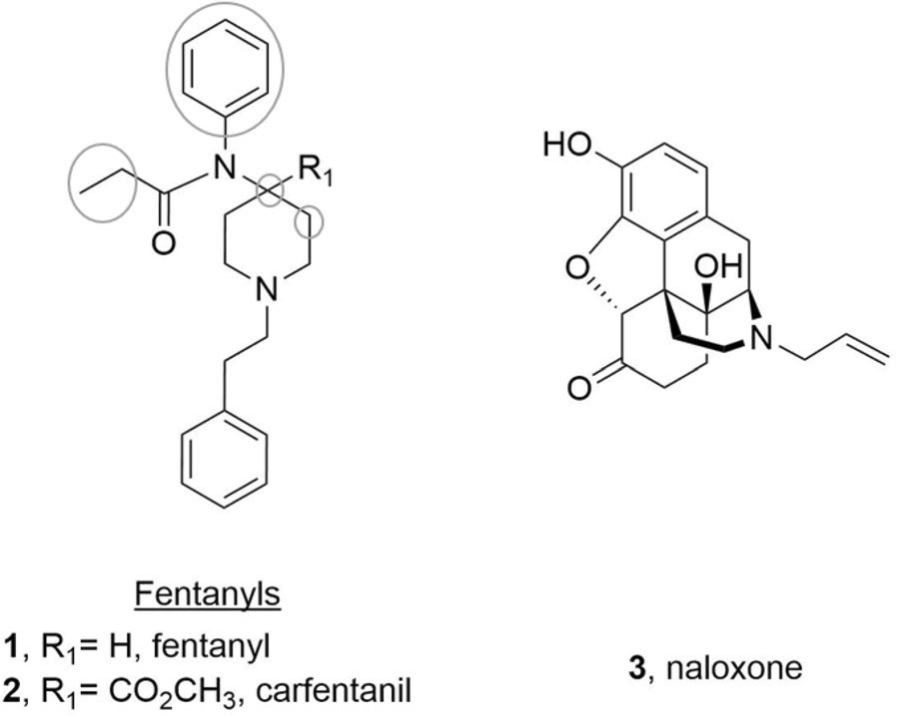
Structures of fentanyls and naloxone. Each circle represents positions which have been abundantly reported to generate illegal fentanyls via chemical modification with various substituents.

Over the past 5 years, the opioid overdose epidemic in the United States^1^ has been exacerbated by the rise in illicit synthetic opioids such as fentanyl (**1**) and its derivatives (fentanyls, Fig. 1)^2–4^. Indeed, in the 12 months preceding March 2021 alone, the CDC estimated a total of 63,075 synthetic opioid overdose deaths, a 54.5% increase from the previous year^5^. This persistent increase in fentanyls-related overdose deaths reflects these synthetic compounds’ highly rewarding effects, potency in inducing respiratory depression, and widespread availability driven by ease of production and distribution^6–8^. Thus, such challenges are forcing health care providers to reconsider their strategies for treating overdoses caused by synthetic opioids^9–11^.

Naloxone (**3**, NLX) is the most effective clinically available medication to reverse opioid-induced overdoses (Fig. 1)^6,12^. It is a potent antagonist (K_d_ = 0.73 nM^13^) of the mu-opioid receptor (MOR), which is the target underlying opioid induced analgesia, reward, and respiratory depression^14,15^. Parenteral (0.4 mg, 2 mg) and intranasal (IN, 2 mg, 4 mg) NLX formulations are available over the counter in most states for opioid overdose reversal. However, there are growing concerns that those options may be ineffective in reversing overdoses of highly potent synthetic opioids such as fentanyl (K_i_ = 0.39 nM)^16^ and carfentanil (**2**, CFN, K_d_ = 0.08 nM)^17^. Additionally, the relatively short duration of NLX’s action may be insufficient for preventing re-narcotization after overdose with longer-lasting fentanyls. In fact, Tomassoni et al. noted, “Some patients required doses of the opioid antidote naloxone exceeding 4 mg (usual initial dose = 0.1–0.2 mg intravenously), and several patients who were alert after receiving naloxone subsequently developed respiratory failure”^18^. Multiple NLX injections are sometimes required to maintain adequate breathing following re-narcotization after initial NLX treatment^19^. Thus, a better understanding of NLX’s blockade of the MOR over time would help to improve our clinical guidelines regarding NLX administration and dosing for overdose reversal from fentanyls.

Positron emission tomography (PET) and the MOR radioligand, [^11^C]CFN have been used to measure blockade of MOR by NLX non-invasively in humans^20^ and non-human primates^21^. Most [^11^C]CFN PET studies have assessed receptor occupancy (RO) within 10 min after an acute NLX dose for various administration methods^19,22–30^. In particular, two relevant clinical reports have been published on NLX’s clearance rate from MORs. One used a dual coincidence detector system to compare intravenous (IV) NLX with IV nalmefene^31^; the other used PET to obtain RO at two time points after IN NLX^32^. In the current study, we used PET to measure the displacement of [^11^C]CFN binding by IV NLX in the rodent brain. Susequently, we obtained RO at multiple time points to characterize the clearance profile over 2.5 h following IV NLX. For both sets of experiments, we compared two clinically relevant IV NLX doses (0.035 and 0.17 mg/kg), which correspond to human equivalent doses (HED) of 0.4 mg and 2.0 mg, respectively.

## Results

### [^11^C]CFN radiosynthesis and administration

Averaged radiochemical yield and molar activity at the end of the bombardment were 50 ± 12% and 1172 ± 938 GBq/μmol, respectively. The average molar activity and injected CFN mass at the time of [^11^C]CFN injection were 226.4 ± 162.4 GBq/μmol and 60.4 ± 55 ng/kg, respectively. The HPLC analysis confirmed high radiochemical purity (> 99%). There was no significant change in rodent physiological parameters measured during the experiments.

### [^11^C]CFN binding displacement by intravenous NLX

In [^11^C]CFN PET scans, the thalamus showed high uptake and specific binding, so it was used as the region of interest (ROI) to quantify specific binding and RO by NLX^33^. The cerebellum showed fast [^11^C]CFN clearance, so it was used as a reference region (SI Fig. 1A). Average clearance half-time (t_1/2_) of [^11^C]CFN from peak uptake levels was 41.84 min for the thalamus and 7.33 min for the cerebellum. IV NLX pretreatment at 5 min prior to [^11^C]CFN injection reduced [^11^C]CFN uptake in the thalamus to the level of uptake seen in the cerebellum (SI Fig. 2). This provides preclinical evidence suggesting that currently approved doses of IV NLX can abolish specific binding of [^11^C]CFN, and are likely, at peak levels, to temporarily occupy nearly all MORs in the brain.

**Figure 2:**
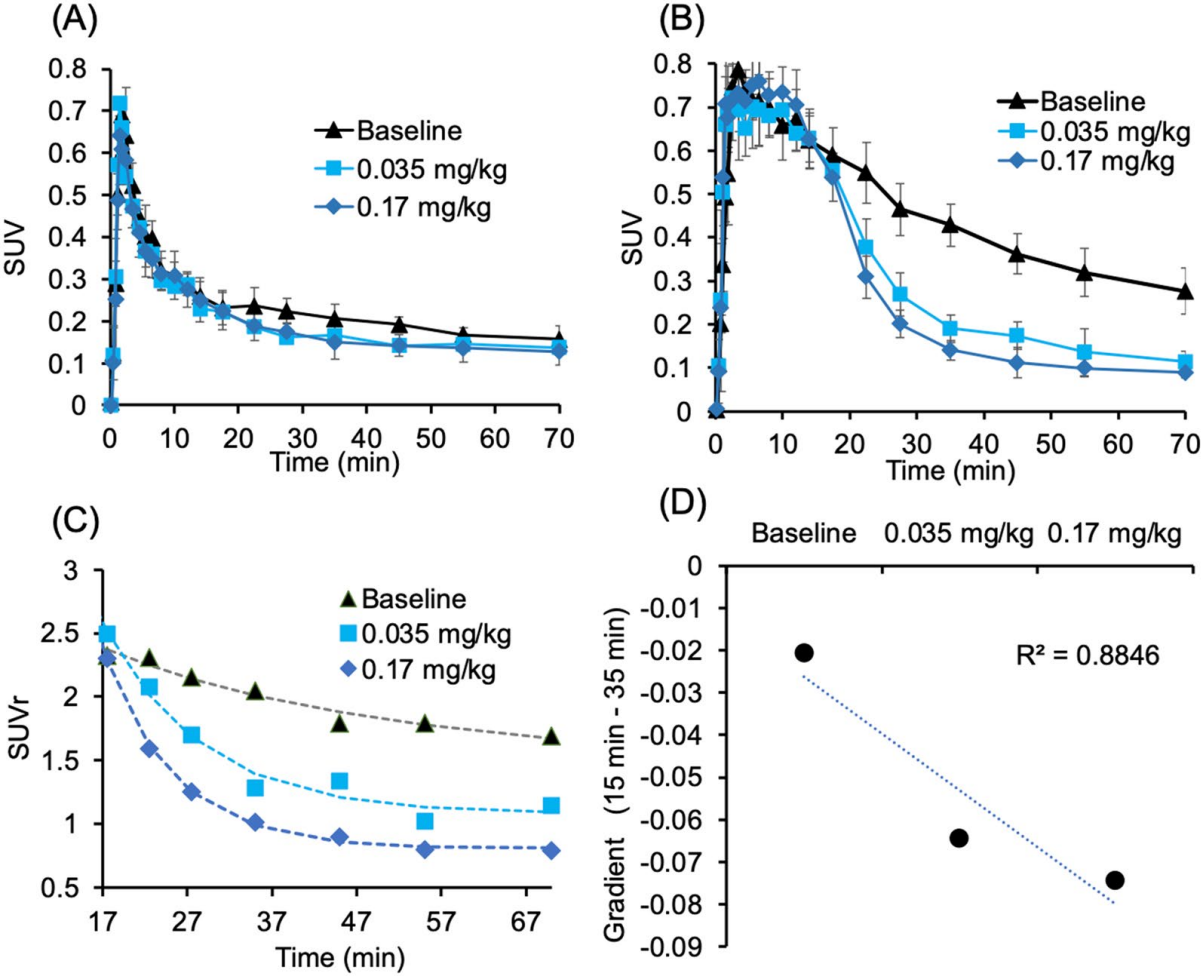
[^11^C]CFN displacement study with two doses of IV NLX (baseline, n = 3; 0.035 mg/kg, n = 3; 0.17 mg/kg, n = 3). Averaged time-activity curves in the cerebellum (**A**) and thalamus (**B**) were generated in standard uptake value (SUV, g/mL). Averaged SUVs of thalamus to cerebellum ratios (SUVr) for baseline and the two NLX doses (**C**). Dose-dependent displacement rate was expressed as gradient change after NLX post-treatment (**D**). NLX was administered at 15 min after [^11^C]CFN injection.

**Figure 3:**
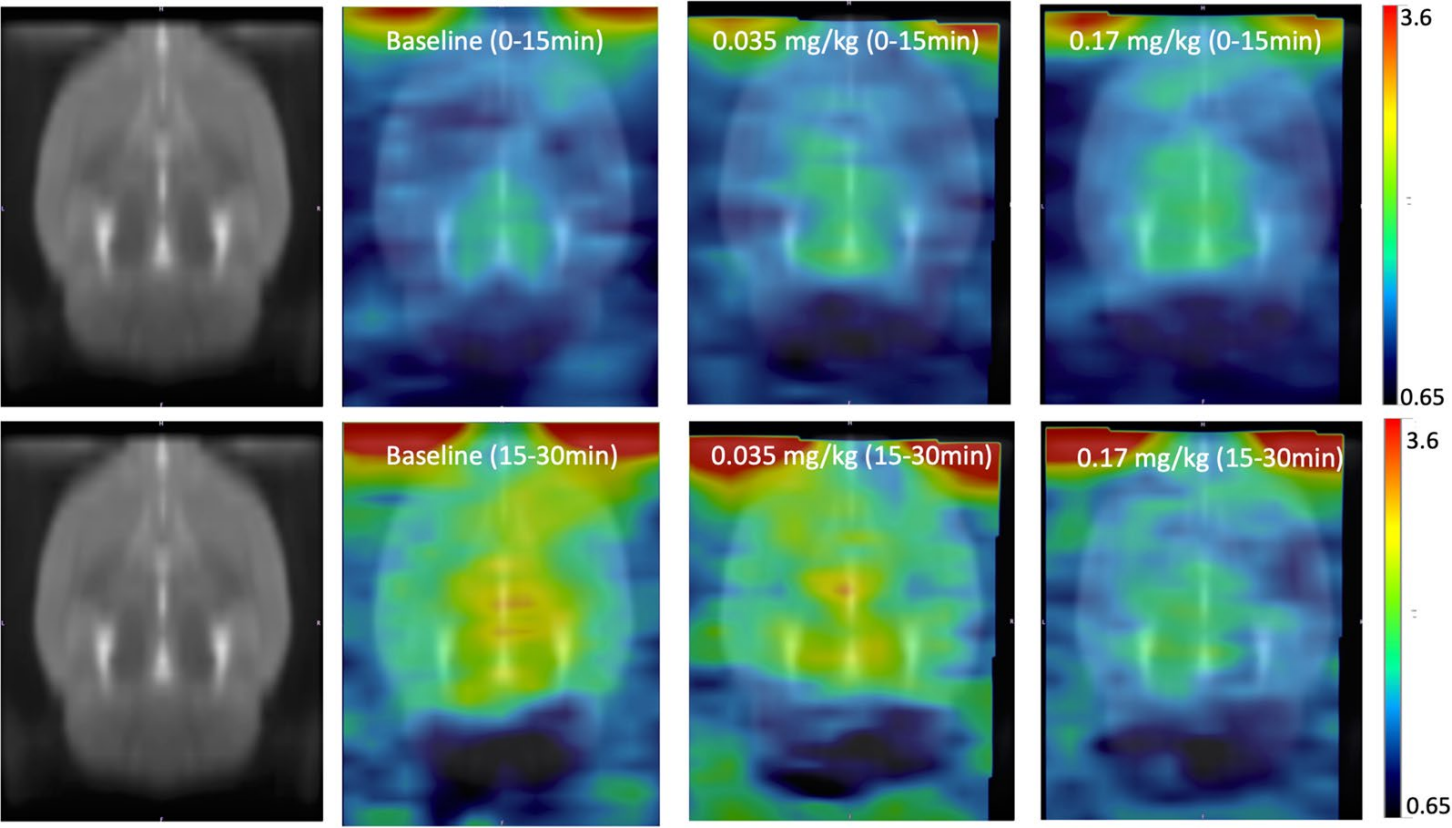
Averaged PET brain images of [^11^C]CFN for baseline and the NLX treatments (0.035 mg/kg and 0.17 mg/kg NLX) aligned with W. Schiffer Rat Brain atlas using PMOD. NLX was given 15 min after [^11^C]CFN administration to assess displacement. The top row corresponds to the average images obtained at 0–15 min and the bottom row corresponds to averaged images obtained at 15–30 min, in which at 15 min, NLX was injected at 0.035 and 0.17 mg/kg doses.

To characterize NLX’s displacement of [^11^C]CFN at MORs, NLX administration was given at 15 min after [^11^C]CFN injection. Both doses of NLX gradually diminished [^11^C]CFN binding in the thalamus to the level observed in the cerebellum (reference region), but the higher dose displaced it faster than the lower one (Figs. 2 and 3). Time-activity curves of [^11^C]CFN in the thalamus prior to NLX injection did not differ statistically between the control and the NLX treatment groups (Student’s *t* test p = 0.14 for 0.17 mg/kg; p = 0.31 for 0.035 mg/kg) (Fig. 2B). After the IV NLX challenge, the time to reach equivalent levels of [^11^C]CFN uptake in the thalamus and cerebellum (ratio of standard uptake value, SUVr = 1) was 62 ± 9 min for the lower NLX dose (0.035 mg/kg) and 34 ± 4 min for the higher dose (0.17 mg/kg), and the difference between the two doses was significant (p = 0.0095, Student’s *t* test) (Fig. 2C). Estimation of the gradient change after NLX injection^34^, showed that NLX’s displacement of [^11^C]CFN was significantly faster for the 0.17 mg/kg NLX dose than for the 0.035 mg/kg dose (p = 0.002, Student’s *t* test, n = 3) (Fig. 2D).

### Duration of receptor occupancy after pretreatment with IV NLX

**Figure 4:**
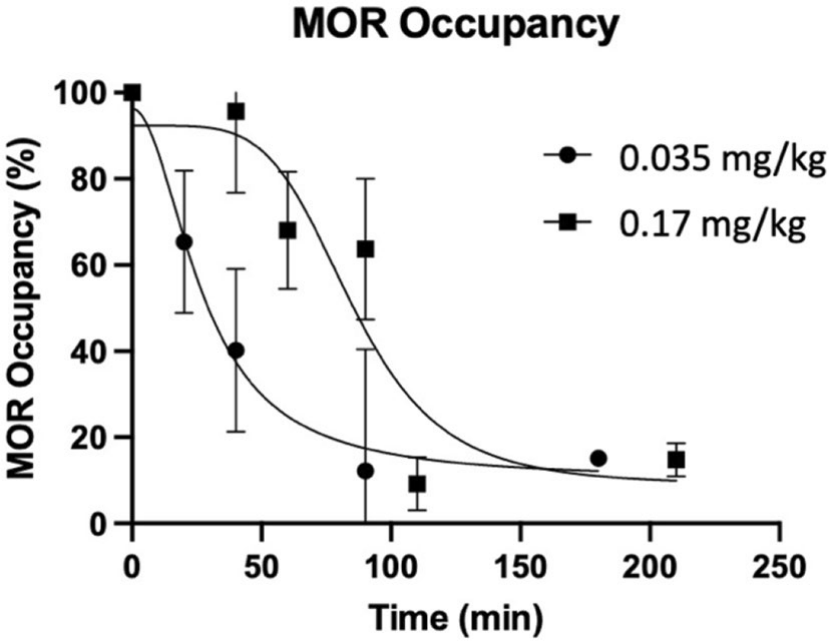
MOR occupancy profiles after IV NLX for two doses (circle, 0.035 mg/kg; rectangle, 0.17 mg/kg). Occupancy data averaged for each time point were plotted with a sigmoidal function. NLX 0.035 mg/kg corresponds to 0.4 mg HED and NLX 0.17 mg/kg to 2 mg HED. Error bars correspond to standard deviations.

**Figure 5:**
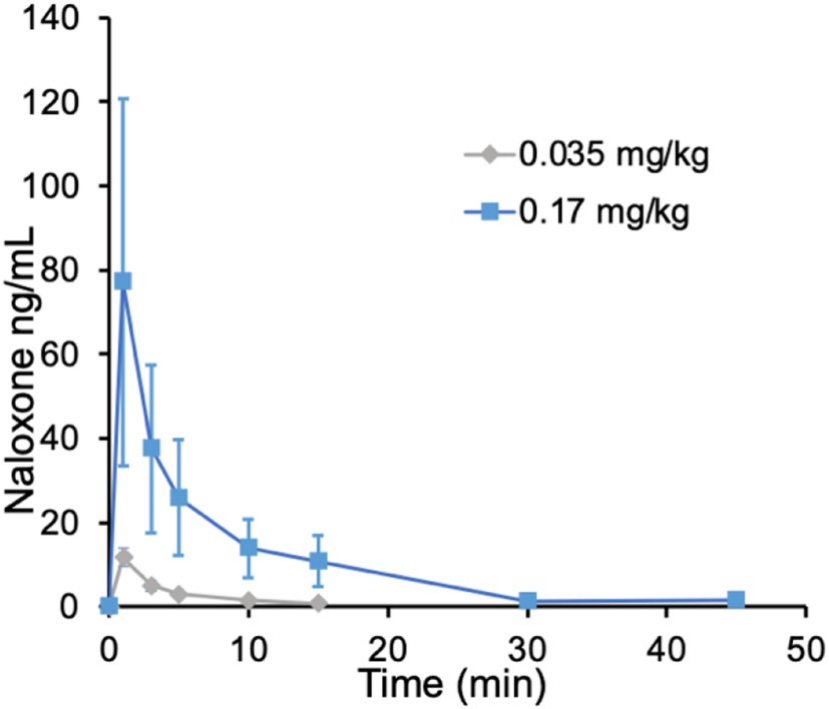
Averaged plasma concentration after IV NLX in rats. NLX was administered intravenously for each of the two doses (0.035 mg/kg, n = 3; 0.17 mg/kg, n = 3).

Rats were pretreated with IV NLX (0.035 mg/kg, 0.17 mg/kg) at various time points before [^11^C]CFN injection. While there were no differences in [^11^C]CFN uptake in the cerebellum for any time points, the uptake of [^11^C]CFN in the thalamus differed significantly between the various time points of NLX pretreatment, showing a gradual recovery towards baseline over two hours. The apparent specific binding measurement ($SUV_{r} - 1$) of [^11^C]CFN at baseline (n = 8) and at various NLX pretreatment times are summarized in Supplemental Table 1. The higher dose of IV NLX (0.17 mg/kg) blocked [^11^C]CFN binding in the thalamus longer than the lower dose (0.035 mg/kg). Specifically, 90 min after IV NLX injection, the averaged RO of 0.17 mg/kg NLX was significantly higher than for 0.035 mg/kg NLX (60% vs 12% RO, p = 0.02) (Fig. 4). Consistently, the clearance half-time of RO for the thalamus was 27 min for 0.035 mg/kg NLX and 85 min for 0.17 mg/kg NLX (Fig. 5).

### Pharmacokinetics assessment of IV NLX

The measured peak plasma NLX concentration differed significantly between the two doses (0.17 mg/kg: 77 ng/mL; 0.035 mg/kg: 1.7 ng/mL; Student’s *t* test, p = 0.01) (Fig. 5). Table 1 shows the estimated pharmacokinetic data for IV NLX in plasma based on a non-compartmental analysis. The average elimination rate of IV NLX (K_e_) was 16 min for 0.17 mg/kg and 7 min for 0.035 mg/kg NLX, respectively.

**Table 1:** Plasma pharmacokinetics of NLX for two doses (0.035 and 0.17 mg/kg, IV) in healthy rats. Values correspond to means (standard deviations).

Dose (mg/kg)	Half-life (min)	Tmax (min)	Cmax (ng/mL)	AUC (0–inf)
0.035 (n = 3)	7.3 (3.5)	6.8 (3.3)	12.4 (1.7)	53.2
0.17 (n = 3)	16 (5.8)	15.0 (5.0)	50.5 (1)	489.6

### Correlation between receptor occupancy and plasma NLX concentration

NLX plasma concentration decreased rapidly for the two doses of IV NLX, but lasted longer for the higher dose (SI Fig. 3). The plasma NLX levels necessary to achieve half-maximal receptor occupancy (EC_50_) were estimated to be 0.2 ng/mL as assessed by fitting the association between NLX RO and plasma NLX concentrations (SI Fig. 4). NLX RO levels plateaued at 90% for NLX plasma concentrations higher than 1.5 ng/mL.

## Discussion

Highly potent fentanyl derivatives are the main contributors to the steep rise in opioid overdose mortality. To cope with the challenges in reversing overdoses caused by potent fentanyls, the Food and Drug Administration (FDA) recently approved a high dose of IN NLX (8 mg^35^) and a 5 mg NLX injection dose^36^. Balancing the risks and benefits of increasing NLX doses used for opioid reversal is a topic of clinical interest^37^. Thus, understanding the relationship between NLX’s dose and its onset and duration of MOR blockade will play a key role in developing proper guidelines for fentanyls overdose reversal.

In the current study, we used clinically relevant doses of IV NLX to characterize its efficacy in displacing [^11^C]CFN in the male rat brain. Moreover, we quantified the rate and duration of MOR occupancy by IV NLX over time. We showed that [^11^C]CFN clearance rate by IV NLX was dose-dependent, with the higher dose displacing CFN more quickly than the lower dose. Specifically, the clearance time to reach half maximal [^11^C]CFN specific binding in the thalamus for the 0.17 mg/kg NLX (HED 2 mg) dose was 21.74 min, significantly faster than 27.05 min for the 0.035 mg/kg NLX (HED 0.4 mg) dose. This result would therefore suggest that higher NLX doses are likely to exhibit faster onset of action in overdose reversal settings than lower NLX doses.

The higher NLX dose also resulted in a longer duration of MOR occupancy than the lower NLX dose. We built up RO profiles by performing [^11^C]CFN PET studies at multiple time points after NLX administration separately for each of the two doses. Half time of RO clearance was 27 min for the 0.035 mg/kg NLX dose, whereas it was 85 min for the 0.17 mg/kg dose, showing that the duration of MOR blockage by NLX is dose dependent. The duration of greater than 80% MOR blockade for the 0.17 mg/kg dose was five times longer compared to that of the 0.035 kg/mg dose. As shown in Fig. 3, RO of less than 30% occurred within 50 min for the lower dose (0.035 mg/kg), whereas it occurred at 100 min for the higer dose (0.17 mg/kg). The fast RO clearance observed with IV NLX could explain why multiple NLX doses are often required to prevent re-narcotization following overdoses with fentanyls^38^.

In a separate group of rats, NLX pharmacokinetics were measured in plasma for the two NLX doses and to correlate them with levels of RO, we estimated that the plasma NLX concentration for half-maximal receptor occupancy (EC_50_) was low (0.2 ng/mL). However, it is likely that the NLX concentration in the brain was much higher than in the plasma (SI Fig. 3) due to NLX’s high lipophilicity and the short-lasting peak in plasma after IV administration. Future studies are required to assess the relationship between plasma and brain NLX levels and to ascertain what level of RO is needed to restore and sustain proper breathing following fentanyls overdoses.

Previously, Kim et al. measured [^11^C]CFN uptake in the human brain at four time points over 9 h using a dual coincidence detector system. They reported that the clearance half-time of RO by IV NLX (2 mg) was 2 ± 1.6 h after administration^31^. These data, in conjunction with our own, suggest that 2 mg IV NLX might be inadequate for preventing re-narcotization after overdose with long half-life fentanyls (e.g., CFN T_1/2_ = 7.7 h). Similarly, Johansson et al.^32^ used PET to measure the specific binding of [^11^C]CFN in healthy human brains at two separate time intervals (0–60 min, 300–360 min) after administration of 2 mg IN NLX. They found that the clearance half-time of RO occurred 2–3 h after NLX administration and that the duration of NLX’s RO at MORs was significantly longer for the 4 mg dose than the 2 mg dose. Thus, both preclinical and clinical studies provide evidence that may justify the use of higher doses of NLX, thereby corroborating the FDA’s recent decision to approve higher NLX formulations^35,36^. Despite these conclusions, there are serious drawbacks for using higher doses of NLX, namely, NLX-precipitated withdrawal symptoms. Likewise, the use of potent MOR antagonists with a longer duration of action, such as naltrexone and nalmefene, has been proposed^39^, but concerns of protracted opioid withdrawal need to be considered. In this respect, it would be valuable to determine the minimal levels of RO needed to sustain normal breathing and cardiovascular function following an overdose.

### Limitations

Our study is limited by species differences in both the NLX pharmacokinetics under anesthesia, and pharmacodynamics of opioid induced respiratory depression. Since the acquisition time for a single PET scan requires at least 1 h, rapid RO change induced by NLX could not be accurately measured by conventional binding potential estimation. Additionally, it was challenging to predict RO with plasma NLX concentrations over time because NLX’s clearance rate in plasma is very fast. Although our displacement studies showed prompt and very effective displacement of [^11^C]CFN binding by IV NLX, experiments used tracer doses of CFN and pharmacological doses of fentanyls may exhibit slower displacement rates. Our study, as well as prior studies, evaluated NLX’s pharmacokinetics of [^11^C]CFN displacement and RO in animals or individuals who were physiologically stable, whereas opioid overdoses subjects may have complications in cardiovascular function, which could jeopardize NLX bioavailability and delivery to the brain. Finally, after surveying all previous NLX RO studies, we point out that there is little information on what level of RO is effective for opioid overdose reversal, which would be valuable to provide clear guidelines for clinical therapeutics.

## Conclusion

Using two clinically relevant doses of IV NLX, we documented fast and effective displacement of [^11^C]CFN binding but short lasting MOR occupancy in the rodent brain. The effects were dose dependent such that a higher dose of NLX displaced CFN faster and had longer duration of MOR blockade. Our results indicate that higher initial doses of NLX could more quickly revert an overdose and that repeated doses could help prevent re-narcotization from these long-acting fentanyls.

## Materials and methods

All rat studies were approved by the Clinical Center Animal Care and Use Committee of National Institutes of Health (protocol number, NIAAA 19-01) and complied with the Guide for the Care and Use of Laboratory Animals. The study was designed in accordance with ARRIVE guidelines. [^11^C]CFN PET studies were performed in male Long Evans rats (n = 63) using a small animal PET scanner (MicroPET Focus 220, Siemens). For the displacement study, nine rats underwent PET scans (290.3 ± 26.4 g, Charles River Laboratories), while for the RO study, 48 rats were used (310.7 ± 72.3 g, Charles River Laboratories). Six rats were performed for the plasma pharmacokinetic analysis (225.5 ± 28.6 g, Charles River Laboratories).

Animals were anesthetized with isoflurane (Forane, Baxter Healthcare) using an anesthesia machine (SurgiVet VaporStick, Smiths Medical) and vaporizer (SurgiVet 100 Series, Smiths Medical). Vitals (heart rate, respiratory rate, spO_2_, and temperature) were monitored using a pulse oximeter and heart rate monitor (MouseSTAT, Kent Scientific). A heat lamp was used to maintain body temperature (Model# 51152, Brandt Industries). Tubing for catheters (BTPE-10 for infusion, BTPU-27 for blood withdrawal) and other surgical materials were obtained from Instech Laboratories. Bolus [^11^C]CFN injections were performed using a syringe pump (PHD 2000, Harvard Apparatus), while bolus plus constant infusion (B/CI) injections were performed using a programmable pump (Pump 11 Elite, Harvard Apparatus). Blood plasma was obtained by centrifugation (MiniSpin, Eppendorf). [^11^C]CFN was synthesized according to the reported procedure with minor modifications^40,41^ (Supplementary materials).

### Rodent PET studies

Anesthesia in rats was initially induced with isoflurane (5.0%) in oxygen for 5 min and then was maintained at a lower level of isoflurane (1.5–2.5%), monitoring vitals throughout the experiments. Catheters were placed in the left femoral vein for [^11^C]CFN injection. For displacement studies, [^11^C]CFN was administered as a bolus (1 min), followed by IV NLX 15 min later. For RO studies, [^11^C]CFN was administered via a B/CI method (K_bol_ = 80 min) that lasted the entire duration of each scan. Before radiotracer injection, rats were pretreated with IV NLX at selected time points (0.035 mg/kg: 20, 40, 60, 87, 180 min; 0.17 mg/kg: 40, 60, 90, 110, 210 min). List-mode data was acquired over 80 min after a 10 min transmission scan with a Co-57 point source for attenuation correction. PET data was reconstructed into 22 frames (6 × 20 s, 5 × 60 s, 4 × 120 s, 3 × 300 s, 3 × 600 s, and 1 × 1200 s) using filtered back-projection. The average activity injected was 14.0 ± 8.6 MBq and the average CFN mass injected was 60.4 ± 55 ng/kg.

### PET imaging processing and tracer kinetic analysis

Time-activity curves were obtained as standard uptake value (SUV, g/mL) using PMOD (3.807). Two regions of interest (ROIs) were analyzed for [^11^C]CFN uptake: the thalamus due to its high concentration of MORs^42^ and high specific binding, and the cerebellum, which was used as a reference region mostly devoid of specific binding^43^.

The ROI template was drawn using anatomical information extracted from a [^18^F]FDG PET scan obtained for this purpose following a [^11^C]CFN scan in one rat. ROIs were drawn in the cerebellum and thalamus, avoiding border regions, and were applied to generate time-activity curves.

SUVr was calculated for each frame as the ratio between thalamic and cerebellar SUVs. For RO studies, the value of $SUV_{r} - 1$ was used as an apparent specific binding measurement. B/CI method achieved constant radioactivity levels in the ROIs and in the reference region^44,45^ and consistent with this we obsereved that activity curves were plateaued after 15 min. Therefore, the value of SUVr was calculated directly from the concentration ratio of thalamus to cerebellum (15–40 min). Receptor occupancy [RO(%)] was calculated using Eq. (1)^46^:

1 $$RO\;(\% ) = 100 \times \frac{{baseline\; (SUV_{r} - 1) - post\, drug \;(SUV_{r} - 1) }}{{baseline \;(SUV_{r} - 1)}}.$$

### Plasma pharmacokinetics assessment of IV NLX

To determine plasma concentrations of NLX over time, a NLX bolus was administered to 6 male rats (225.5 ± 28.6 g; n = 3, 0.035 mg/kg; n = 3, 0.17 mg/kg) via penile vein, and arterial whole blood samples (250 μL) were collected at 0, 1, 3, 5, 10, 15, 30, 45, 60, and 90 min after NLX injection. Each blood sample was centrifuged at 14,500 RPM for 3 min to give each plasma sample, followed by immediate freezing on dry ice until stored at − 80 °C. Plasma NLX concentration was determined using LC–MS/MS (Bioanalytical Shared Resource Laboratory, Virginia Commonwealth University School of Pharmacy), with a detection limit of NLX of 1 ng/mL. Pharmacokinetics parameters were estimated by non-compartmental analysis and plasma curves were fitted using two exponential clearance model.

Given the plasma concentration, *C*_*u*_, and the K_d_ values for the MOR, RO was calculated according to the reaction kinetics between a MOR and NLX, as follows^47^:

$$Occupancy\;(\% ) = \frac{{C_{u} }}{{C_{u} + K_{d} }}.$$

### Statistical analysis

The descriptive statistics and computations for data analysis were performed using MATLAB and Statistics Toolbox Release 2012b, The MathWorks, Inc., Natick, Massachusetts, United States. In all analyses, the statistical significance (alpha level) was set at p < 0.05.

## Supplementary Information

Supplementary Information.

## Data Availability

The datasets analysed during the current study are available from the corresponding author upon reasonable request.
