# Supplementary material for: Naloxone’s dose-dependent displacement of [11C]carfentanil and duration of receptor occupancy in the rat brain
Source: Sci Rep. 2022 Apr 19;12:6429. doi: 10.1038/s41598-022-09601-2 (PMC9018944; doi:10.1038/s41598-022-09601-2)
Supplement: Supplementary file 1 — Supplementary Information. [file 41598_2022_9601_MOESM1_ESM.docx]

Supplement Table 1.

|  | Pretreatment (min) | Number | SUVr-1 | RO |
| --- | --- | --- | --- | --- |
|  |  | of rats | TH | TH |
| Baseline |  | 8 | 1.44±0.32 |  |
| 0.035 mg/kg | 20 | 4 | 0.45±0.22 | 65.36±16.48 |
|  | 40 | 6 | 0.78±0.25 | 40.24±18.93 |
|  | 60 | 2 | 0.60±0.08 | 53.98±5.95 |
|  | 87 | 2 | 1.15±0.37 | 12.23±28.24 |
|  | 180 | 3 | 1.11±0.02 | 15.11±1.14 |
| 0.17 mg/kg | 40 | 4 | 0.06±0.24 | 95.74±18.93 |
|  | 60 | 4 | 0.35±0.15 | 73.03±11.23 |
|  | 90 | 8 | 0.47±0.21 | 63.70±16.33 |
|  | 110 | 4 | 1.05±0.18 | 25.71±17.1 |
|  | 210 | 2 | 1.11±0.05 | 14.82±3.84 |

Supplementary Table 1. Pharmacokinetic parameters for NLX doses of 0.035 and 0.17 mg/kg assessed at different pretreatment times. BP=Binding Potential, RO=Receptor Occupancy, TH=thalamus, CB=cerebellum (reference regions).

Supplement figure 1.


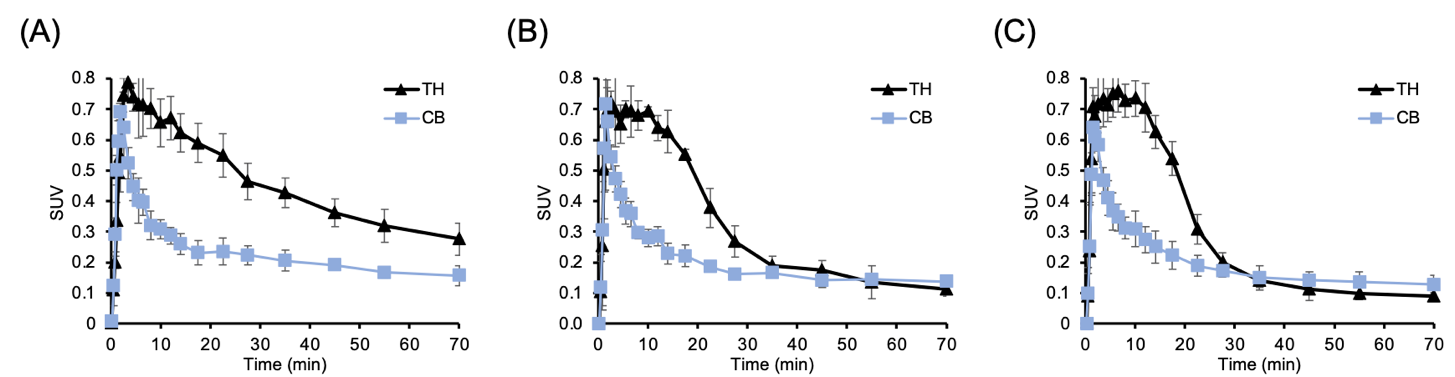


Suppl Figure 1. Averaged time-activity curves of [^11^C]CFN for the baselines (A, n=3), NLX post-treatment scans (B, n=3; IV, 0.035 mg/kg), and NLX post-treatment scans (C, n=3; IV, 0.17 mg/kg). NLX was administered at 15 min after [^11^C]CFN injection. [^11^C]CFN uptake is expressed as standard uptake values (SUV) in the thalamus, (TH), which was the target region for its high specific binding and in the cerebellum (CB), which served as a reference region devoid of specific binding.

Supplement Figure 2.


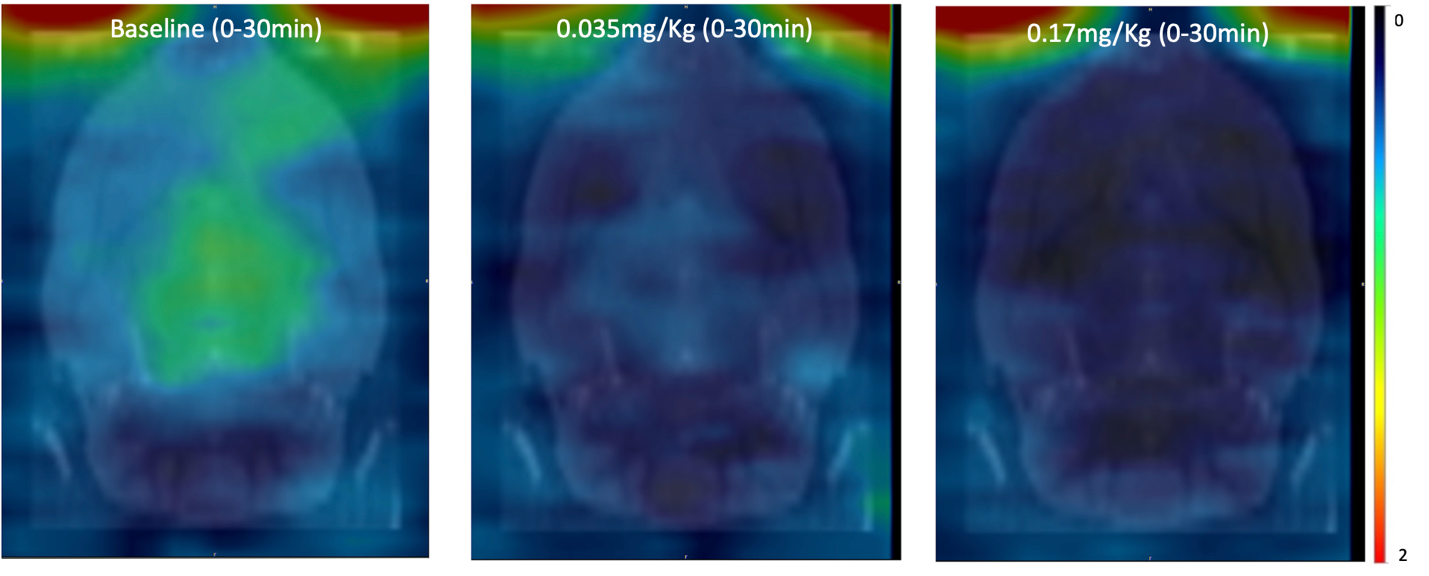


Suppl Figure 2. PET brain images of [^11^C]CFN for baseline and the NLX pretreatments (0.035 mg/kg and 0.17 mg/kg NLX). NLX was given 5 min prior [^11^C]CFN administration to assess blocking MORs.

Supplement Figure 3.


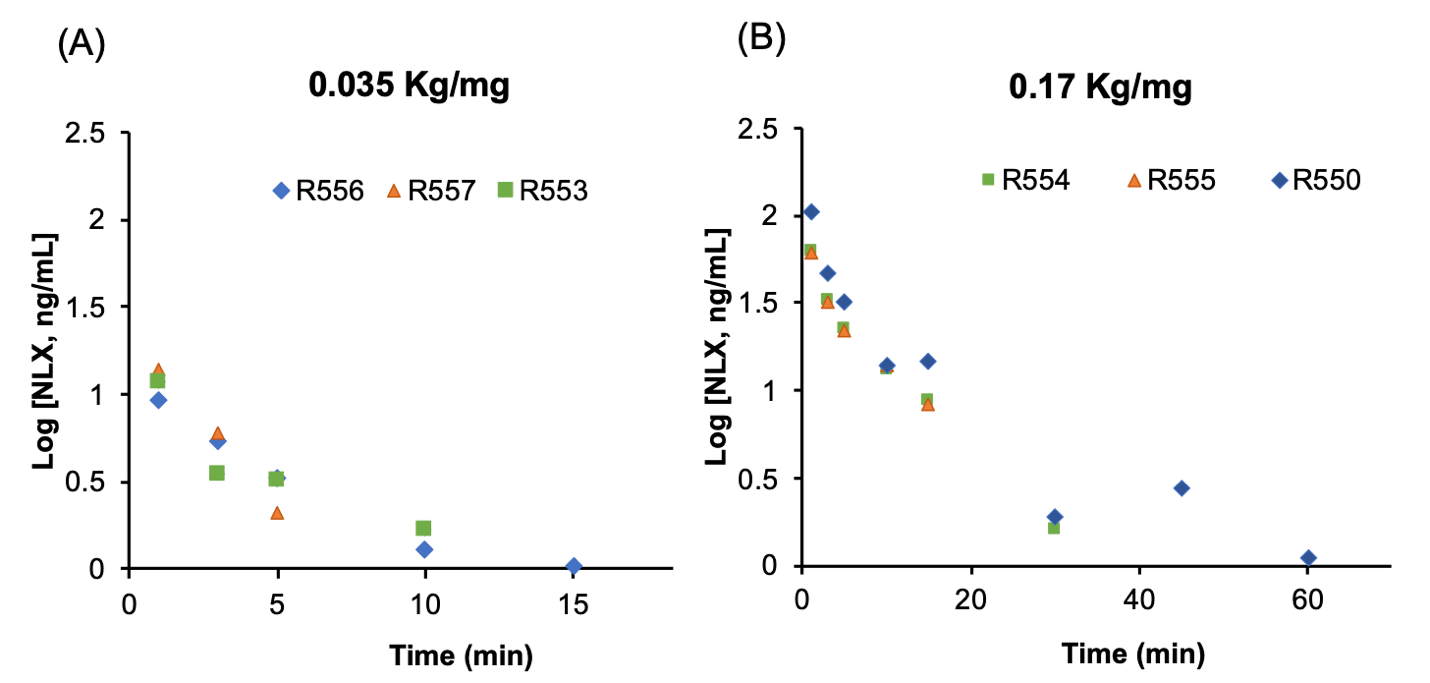


Suppl Figure 3. Plasma concentration of naloxone in individual rats for the two doses of IV NLX (A, 0.035 mg/kg, n=3; B, 0.17 mg/kg, n=3).

Supplement Figure 4.

Suppl Figure 4. Correlation between MOR occupancy and IV NLX concentration in plasma. Estimated half-maximal effect (EC_50_) is 0.2 ng/ml.

Synthesis of [^11^C]Carfentanil ([^11^C]**CFN**)

The precursor for [^11^C]CFN, Desmethylcarfentanil acid, was purchased from American Biochemicals (College Station, TX, USA). All other chemicals were purchased from Millipore Sigma and used without any further purification. No-carrier-added [^11^C]CO_2_ was generated by nuclear reaction ^14^N(p,α)^11^C, bombarding a nitrogen gas target containing 1% oxygen with a proton beam using a cyclotron (PETrace, GE). Conversion to [^11^C]CH_3_I and [^11^C]methylation were performed using FX-MeI and FX-M automated synthesizers (GE Healthcare, Chicago, IL USA), respectively.

Anhydrous DMSO (200 µL) solution containing desmethylcarfentanil acid (1 mg, 2.64 µmol), Cs_2_CO_3_ (2 mg, 6.14 µmol) was vortexed for 1 min. After [^11^C]methyl iodide was transferred in a stream of helium at room temperature, the reaction mixture was heated at 120°C for 3 min. Crude mixture was purified with semi-preparative HPLC with a monolithic column (10x100 mm, Onyx Monolithic C18, Phenomenex; flow rate, 5 mL/min; eluent, 0.01M phosphate buffer/ethanol=51.5/48.5; pH = 7.2-7.4; UV wavelength, 218 nm; retention time, 8 min). The collected portion of [^11^C]CFN was adjusted to contain less than 10% ethanol content for injections. For quality control, analytical HPLC analysis was performed using an Agilent 1100 system equipped with a ZORBAX Eclipse XDB C18 column (4.6x150mm, Agilent, Santa Clara, CA), monitoring for absorbance at 218 nm and radioactivity using a flow count radioactivity detector (Carroll Ramsey and Associates, Fort Collins, CO, USA). [^11^C]CFN was eluted at a flow rate of 1.0 mL/min (retention time, 5 min) with an isocratic solvent mixture (water/acetonitrile, 60/40) containing trifluoroacetic acid (0.1%).
